# Confirmation of biallelic *VPS11* variants as a cause of complex dystonic syndrome

**DOI:** 10.1016/j.prdoa.2025.100419

**Published:** 2025-12-31

**Authors:** Arnaud Storck, Marie Thérèse Abiwarde, Gaelle Hardy, Anne-Sophie Lebre, Sophie Scheidecker, Maria Cristina Antal, Mathieu Anheim, Thomas Wirth

**Affiliations:** a Service de Neurologie, Département de Neurologie, Hôpitaux Universitaires de Strasbourg, Hôpital de Hautepierre, 1, Avenue Molière, Strasbourg, Cedex, France; b Service de Neurologie Pédiatrique, Hôpital Universitaire de Strasbourg, Strasbourg, France; c UM de Génétique Moléculaire : Maladies Héréditaires et Oncologie, Institut de Biologie et de Pathologie, CHU Grenoble Alpes, Grenoble, France; d Université Paris Cité, Institute of Psychiatry and Neuroscience of Paris (IPNP), INSERM U1266, [Krebs team], 75014 Paris, France; e Université Reims Champagne Ardenne and CHU de Reims, Reims, France; f Laboratories of Genetic Diagnosis, Institut de Génétique Médicale d’Alsace (IGMA), Strasbourg University Hospitals Strasbourg France, Strasbourg, France; g Université de Strasbourg – Faculté de Médecine, Maïeutique et Sciences pour la Santé Institut d’Histologie - Service Central de Microscopie Électronique, ICube UMR7357 – équipe IMIS Hôpitaux Universitaires - Service de Pathologie - UF6349 Foetopathologie, France; h Institut de Génétique et de Biologie Moléculaire et Cellulaire (IGBMC), INSERM-U964/CNRS-UMR7104/Université de Strasbourg, Illkirch, France

**Keywords:** Dystonia, Spastic paraplegia, Neurogenetic, Endolysosomial pathway

## Abstract

• *VPS11* is associated with hypomyelinated leukodystrophy affecting children.
• A single case of adult-onset generalized dystonia was reported.
• We provide the first replication by describing a second case of complex dystonia related to biallelic variants in *VPS11*.

While acknowledged as a cause of hypomyelinated leukodystrophy [1,2], *VPS11*-associated dystonia was described in a single patient [3].

We report here a case of *VPS11*-associated dystonia concerning a female patient born at term from a consanguineous union without family history of neurological disorders. Her psychomotor acquisitions were normal until the age of 12 when she developed repeated episodes of paroxysmal gait disturbances lasting few minutes and triggered by exercise. These manifestations halted spontaneously before recurring at the age of 17. Cerebral and medullar MRI were unremarkable. The phenotype subsequently broadened with the occurrence of left-sided myoclonus, pyramidal syndrome of four limbs and cervical dystonia **(**Video**)**. Levodopa was initiated in the hypothesis of a dopa-responsive dystonia without efficacy.

Her condition worsened following an infection causing the exacerbation of movement disorders. A brain MRI identified FLAIR hypersignal located in the right temporal lobe (Fig. 1**)**. EEG revealed background slowing while a first lumbar puncture showed no anomaly. Aciclovir was started in the hypothesis of a herpetic encephalitis and the patient referred to the Strasbourg University Hospital Neurology department. Further examination revealed pyramidal syndrome of four limbs, left-sided hemidystonia, mild cerebellar ataxia, positive generalized asymmetric asynchronous rest and action myoclonus, and dysphagia **(**Video**)**. A second lumbar puncture was unremarkable except for tau-protein elevation. Repeated brain MRI revealed anterior putaminal and caudate nucleus head hyperintensity on diffusion-weighted imaging (Fig. 1**)** showing diminished hyperintensity without full regression at month 30 while a second EEG was consistent with myoclonic status epilepticus (Fig. 1**)**.

**Fig. 1:**
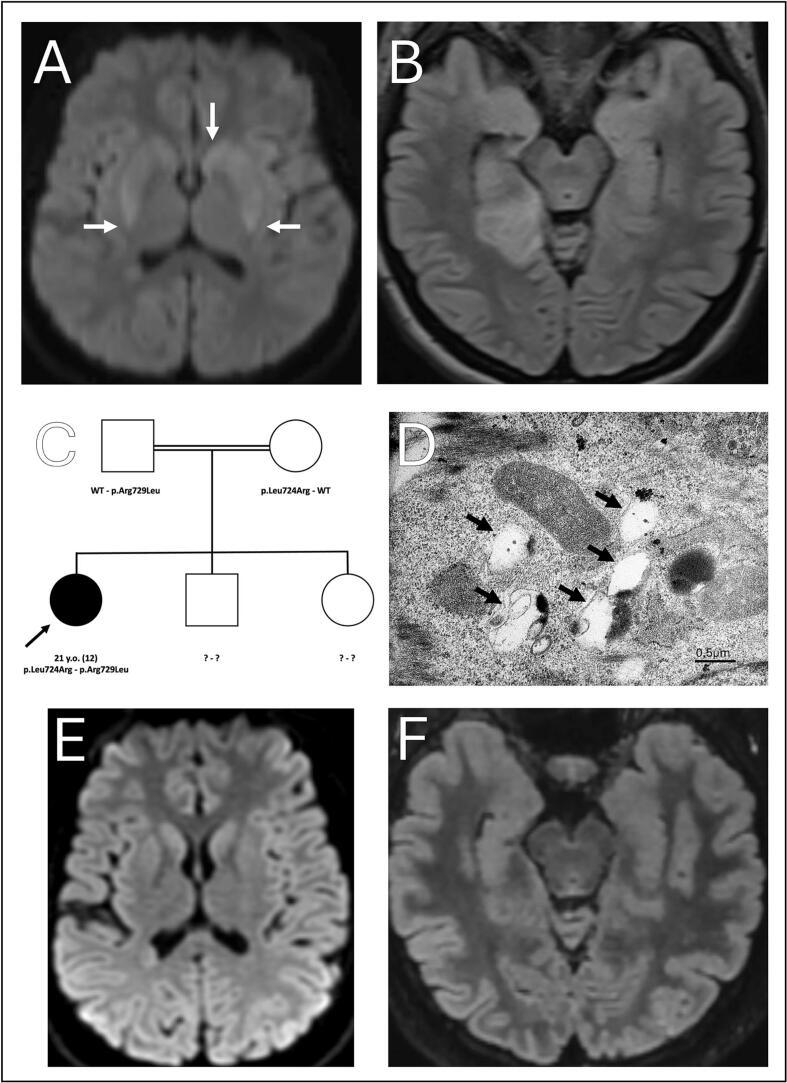
(A.) Diffusion-weighted hyperintensity in the left caudate nucleus and posterior putamens (arrows). (B.) FLAIR hyperintensity in the right mesial temporal lobe. (C.) Familial pedigree. Current age and age at diagnosis (in parentheses). (D.) Electron microscopy of cultured fibroblasts revealing large mainly clear vacuolar structures (arrows) (E.) June 2023 MRI showing regression of previous diffusion-weighted hyperintensities. (F.) June 2023 MRI showing regression of the previous FLAIR hyperintensity.

Empirical treatments (Supplementary material 1) were administered in the hypothesis of autoimmune encephalitis or inborn error of the metabolism, however unsuccessfully, the patient requiring admission to the Pediatric Intensive Care Unit owing to coma. Detailed description of the therapeutics initiated is available as supplementary. The patient subsequently improved in the following months **(**Video**)**.

Her clinical milestones can therefore be summarized with an asymptomatic period ranging from 0 to 12 years old briefly followed with the onset of exercice-triggered paroxysmal movement disorder lasting minutes, a subsequent remission from 12 to 17 years old before the onset of cerebellar ataxia, myoclonus, dystonia and pyramidal syndrome with an acute exacerbation leading to coma before gradual recovery. She presented with cerebellar ataxia and spastic paraplegia when last evaluated at 21 years old.

First tier biological tests were unremarkable and a whole genome sequencing was performed in trio with the parents. This research was made possible through access to the data generated by the 2025 French Genomic Medicine Initiative [4]. The patient was found to be compound heterozygous for two missense variants in *VPS11*. The maternal c.2171 T > G; p.(Leu724Arg) variant was absent from populational database (gnomAD V4.1.0) and predicted pathogenic by various *in silico* prediction tools while the paternal c.2186G > T; p.(Arg729Leu) variant was still absent from populational database but with conflicting *in silico* predictions **(**Fig. 1**,** supplementary material 2**)**. Although homozygous variants would have been expected given the consanguinity of the pedigree, indels up to 50 base-pairs were screened using GATK HaplotypeCaller (v4.1.8.0), deletions between 50 base-pairs and 21 kilo-base-airs using Manta (v1.6.0) and copy-number variants larger than 21 kilo-base-pairs using CNVnator (v0.4.1) without the identification of any other structural nor copy-number variants in dystonia-related genes. Electron microscopy of cultured fibroblast biopsy revealed large mainly clear vacuolar structures similar to that of the patient described in Monfrini et al. **(**Fig. 1**)**. Subsequently, the variants were classified as likely pathogenic (class IV ACMG: PS3 + PM2 + PP3) [5].

Our case suggest *VPS11* variants may be associated with a complex dystonic syndrome. Notable difference compared to the prior report consists in an unalike disease course with a current complicated hereditary spastic paraplegia phenotype and the absence of basal ganglia T2 hypointensity, which may be related to the age difference between the two cases. Deterioration following an intermittent event (hereby consisting in an undocumented infection) bears resemblance to inborn errors of metabolism. Whether this episode represents an aspect of VPS11-related disorder or a coincidental event will require further evidence.

Given the consistency of clinical, molecular and histological data between these independent cases [3], it appears henceforth acceptable to consider that *VPS11*-associated clinical spectrum encompasses both, a white-matter pathology and dystonia.

## Declaration of competing interest

The authors declare that they have no known competing financial interests or personal relationships that could have appeared to influence the work reported in this paper.

## References

[1] Edvardson S., Gerhard F., Jalas C. (2015). Hypomyelination and developmental delay associated with VPS11 mutation in Ashkenazi-jewish patients. J. Med. Genet..

[2] Monfrini E., Zech M., Steel D., Kurian M.A., Winkelmann J., Di Fonzo A. (2021). HOPS-associated neurological disorders (HOPSANDs): linking endolysosomal dysfunction to the pathogenesis of dystonia. Brain J. Neurol..

[3] Monfrini E., Cogiamanian F., Salani S. (2021). A Novel Homozygous VPS11 Variant May Cause Generalized Dystonia. Ann. Neurol..

[4] Abadie C., Abderrahmane A., Abdous O. (2025). PFMG2025–integrating genomic medicine into the national healthcare system in France. Lancet Reg Health – Eur..

[5] Richards S., Aziz N., Bale S. (2015). Standards and guidelines for the interpretation of sequence variants: a joint consensus recommendation of the American College of Medical Genetics and Genomics and the Association for Molecular Pathology. Genet. Med..

