# Supplementary material for: Confirmation of biallelic VPS11 variants as a cause of complex dystonic syndrome
Source: Clin Park Relat Disord. 2025 Dec 31;14:100419. doi: 10.1016/j.prdoa.2025.100419 (PMC12808586; doi:10.1016/j.prdoa.2025.100419)

Variants characterization

Bioinformatics analysis

Data demultiplexing: bcl2fastq®, Illumina®

Alignment and matching: BWA-MEM (0.7.17)

Reference genome: GRCh38.p13 primary assembly (Gencode 32, GCA_000001305.2) with masking of pseudoautosomal regions of the Y chromosome and inclusion of EBV genome (NC_007605). Concatenated lines, optical duplicates labelled with biobambam2 (2.0.89).

Variant calling : SNV and indels up to 50 bp: GATK HaplotypeCaller (4.1.8.0). Deletions between 50 bases and 21 kb: Manta (1.6.0). CNV > 21kb: CNVnator (0.4.1).

Genotyping: GenotypeGVCF from GATK (4.1.8.0) in a single file per family, genocoherence of samples verified

Filters and annotations: Variant Effect Predictor (version 98.3) Curagen and CuteVariant software

- compliant with ACMG recommendations (PMID: 25741868) enhanced by the NGS-DIAG network (19/12/2017).

- supplemented by recommendations from rare disease health networks

Genomic analysis strategy

1. Reference transcripts of genes associated with the test indication (list available on auragen.fr) :
2. Reference transcripts of genes involved in mendelian disorders (prioritized according to clinical situation)
3. Genes not associated with mendelian disorders (candidates on genetic and molecular grounds)
4. Any other subset or genomic region within the limits of available knowledge

For each level: coding sequences and intronic boundaries at 50 bp, then complete gene sequences (5'UTR, promoter, 3'UTR, introns...)

The primary diagnostic target corresponds to autosomes, gonosomes without PAR regions and mitochondrial genome. The “coding” diagnostic target corresponds to the coding sequences (plus or minus 50 bases) referenced in the version of Gencode 32 used in Variant Effect Predictor.

List of missense variants with CADD scores > 15 (after exclusion of variants classified as benign in ClinVar) in the AURAGEN IMR26 « Dystonies ou mouvements anormaux rares du sujet jeune » pre-indication panel (457 genes, last update in February 2025)

| Gene | Transmission | Genomic coordinates | c. | p. | Zygoty | Heredity | CADD |
| --- | --- | --- | --- | --- | --- | --- | --- |
| GRIA3 | XLR | chrX(GRCh38):g.123395043G>C | ENST00000620443.2:c.826G>C | p.Glu276Gln | HTZ | Paternal | 22,4 |
| KCNMA1 | AD,AR | chr10(GRCh38):g.77001557T>A | ENST00000286628.14 : c.2116A>T | p.Met706Leu | HTZ | Maternal | 19,79 |
| CLCN2 | AD,AR | chr3(GRCh38):g.184357233C>T | ENST00000265593.9:c.932G>A | p.Arg311Gln | HTZ | Paternal | 23,9 |
| PDGFRB | AD | chr5(GRCh38):g.150132066C>T | ENST00000261799.9:c.1156G>A | p.Val386Met | HTZ | Maternal | 24,4 |
| UNC13A | UNK | chr19(GRCh38):g.17668139T>C | ENST00000519716.7:c.446A>G | p.Asn149Ser | HTZ | Paternal | 25,3 |
| TH | AR | chr11(GRCh38):g.2165248C>A | ENST00000333684.9:c.1036G>T | p.Ala346Ser | HTZ | Paternal | 24,9 |
| UNC80 | AR | chr2(GRCh38):g.209993378C>T | ENST00000272845.10:c.9190C>T | p.Arg3064Trp | HTZ | Maternal | 26,5 |
| ANKRD28 | UNK | chr3(GRCh38):g.15712220C>T | ENST00000399451.6:c.1103G>A | p.Arg368His | HTZ | Paternal | 23,5 |
| NUP62 | AR | chr19(GRCh38):g.49909227G>A | ENST00000352066.8:c.581C>T | p.Thr194Met | HTZ | Paternal | 17,75 |
| AAAS | AR | chr12(GRCh38):g.53307613G>C | ENST00000209873.9:c.1517C>G | p.Ala506Gly | HTZ | Paternal | 17,85 |
| VPS11 | AR | chr11(GRCh38):g.119078902T>G | ENST00000614944.4:c.2141T>G | p.Leu714Arg | HTZ | Maternal | 29,3 |
| VPS11 | AR | chr11(GRCh38):g.119078917G>T | ENST00000614944.4:c.2156G>T | p.Arg719Leu | HTZ | Paternal | 23,2 |

Patient’s milestones:

0 to 12 years old: asymptomatic

12 years old: paroxysmal movement disorder (exercice-triggered, lasting minutes)

12 to 17 years old: asymptomatic

17 years old: cerebellar ataxia, myoclonus, dystonia, pyramidal syndrome, coma

Current presentation: cerebellar ataxia, spatic paraplegia

Administered medication (in chronological order):

Before clinical worsening leading to admission in critical care:

Methylprednisone 1g daily

Intravenous immunoglobulin 2g/kg

Levetiracetam up to 750mg bid

Clonazepam 1mg/24h

Acetazolamide 250mg bid

Thiamine (vitamin B1) 1000mg daily

Panthotenic acid (vitamin B5) 50mg bid

Pyridoxal phosphate (vitamin B6) 500mg daily

Biotine 10mg bid

Folic acid 10mg daily

Added during hospitalization in critical care:

L-Carnitin 1g bid

Zonisamide 50mg bid

Gabapentin 300mg bid

Clonidine 75$\mu g$ bid

Ketogenic diet

Current medication:

Levetiracetam 1000mg bid

Zonisamide 50mg bid

Clonazepam 0,3mg daily

Folic acid 5mg daily

Electroencephalogram showing bilateral delta activity and predominantly right-sided spikes associated with subcontinuous head jerking.


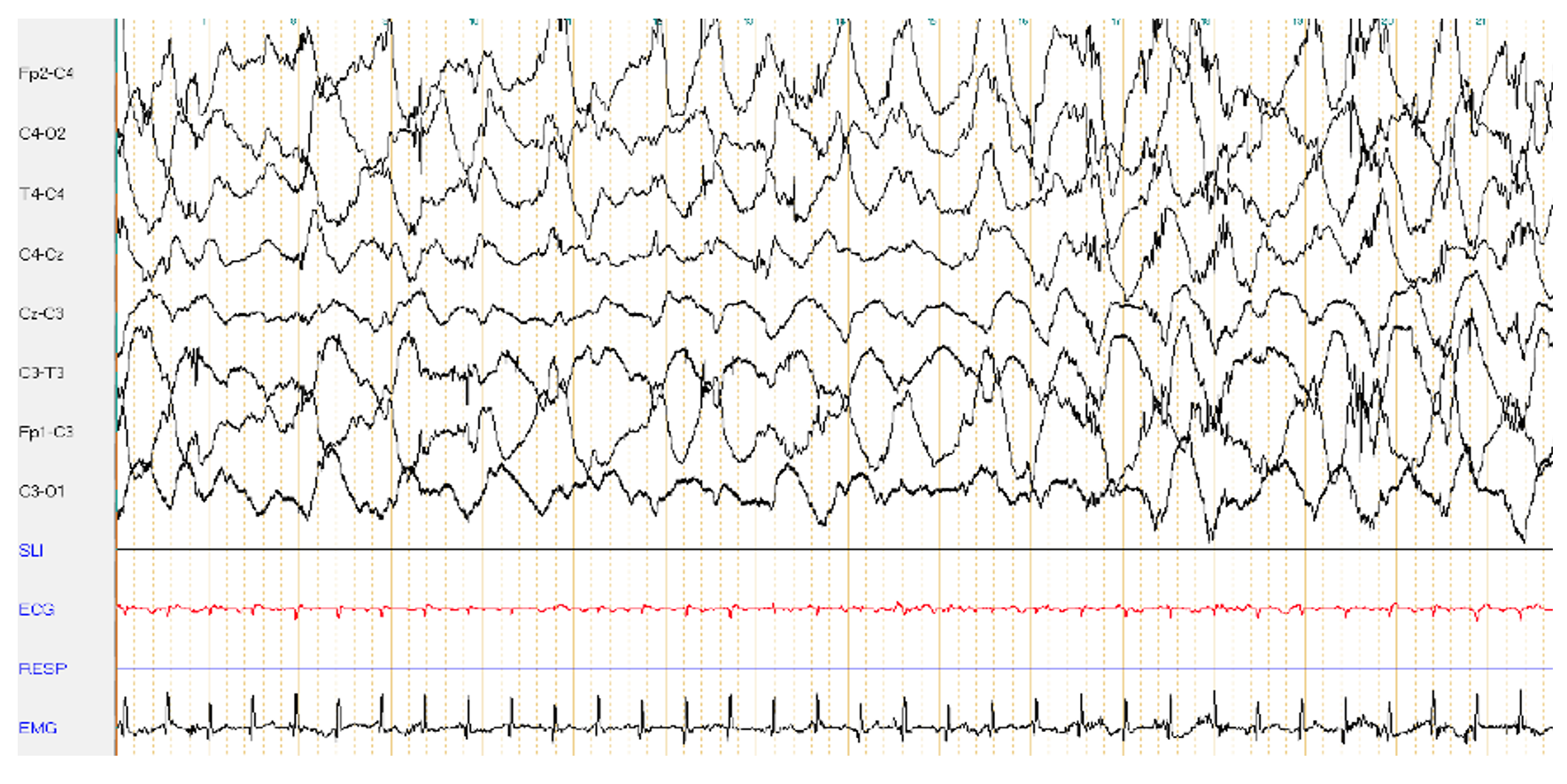

Supplement: Supplementary Data 3 [file mmc3.docx]
